# The Abysmal Organization of Work and Work Safety Culture Experienced by North Carolina Latinx Women in Farmworker Families

**DOI:** 10.3390/ijerph19084516

**Published:** 2022-04-08

**Authors:** Thomas A. Arcury, Sydney A. Smith, Jennifer W. Talton, Sara A. Quandt

**Affiliations:** 1 Department of Family and Community Medicine, Wake Forest School of Medicine, Winston-Salem, NC 27157, USA; 2 Department of Biostatistics and Data Science, Division of Public Health Science, Wake Forest School of Medicine, Winston-Salem, NC 27157, USA; sydnesmi@wakehealth.edu (S.A.S.); jtalton@wakehealth.edu (J.W.T.); 3 Department of Epidemiology and Prevention, Wake Forest School of Medicine, Winston-Salem, NC 27157, USA; squandt@wakehealth.edu

**Keywords:** migrant health, women’s health, occupational health, agricultural health, migrant and seasonal farmworkers, Latinx, organization of work, work safety culture, work safety climate

## Abstract

The occupational health of immigrant workers in the United States is a major concern. This analysis describes two domains, organization of work and work safety culture, important to the occupational health of Latinx women in farmworker families. Sixty-seven Latinx women in North Carolina farmworker families completed a baseline and five follow-up questionnaires in 2019 through 2021. Fifty-nine of the women were employed in the year prior to the Follow-Up 5 Questionnaire. These women experienced an abysmal organization of work and work safety culture. They experienced significant job churn, with most changing employment several times during the 18-month period. Most of their jobs were seasonal, paid less than $10.00 per hour, piece-rate, and almost all without benefits. The women’s jobs had little skill variety (mean 1.5) or decision latitude (mean 1.1), but had high psychological demands (mean 2.0). Work safety climate was very low (mean 13.7), with 76.3% of women noting that their supervisors were “only interested in doing the job fast and cheaply” rather than safely. Women employed as farmworkers versus those in other jobs had few differences. Further research and intervention are needed on the organization of work and work safety culture of Latinx women manual workers.

## 1. Introduction

Immigrant worker health and safety are ongoing concerns in the United States (US) [1]. These workers often are employed in the most hazardous jobs in the most dangerous industries. They are migrant and seasonal farmworkers and chicken catchers in agriculture [2,3,4], line operatives in chicken and meat processing plants [5,6], forestry workers [7,8], fishers [4], landscape and lawn maintenance workers [9,10], residential construction workers [11,12], and hotel and restaurant workers [13,14,15]. Many of these workers are considered essential during the COVID-19 pandemic, with media [16,17] and academic [18,19,20] publications documenting their excessive exposure, morbidity, and mortality compared to the larger US population.

Latinx women constitute a major component of the immigrant workforce [1]. They are vulnerable for the same reasons as are immigrant men: they often lack documentation, lack fluency in English, are poor, have limited educational attainment, and have limited job skills [21,22,23]. In addition, similarly to all women, compared to men they often are paid less for the same work and have limited opportunities for advancement [24]. They are subject to workplace sexual harassment and abuse [25,26,27]. After their hours in paid employment, they have the burden for most domestic responsibilities, including childcare, food preparation, and cleaning [28,29].

The demands on migrant and seasonal farmworkers pose important challenges for occupational justice [2], especially women in farmworker families [23]. These women include those who are themselves employed as farmworkers, as well as those who reside in households in which one or more other adults are employed as farmworkers. Over one million people are employed as migrant and seasonal farmworkers in the US, with 77% identifying as Latinx and two-thirds being immigrants (64% being born in Mexico, 3% in Central America) [30]. Thirty-one percent of farmworkers are women [30].

Several studies have documented important aspects of the health of women in farmworker families, including pregnancy [31,32,33,34], cancer [35], HIV [36], and mental health [37,38,39,40,41]. Other studies have examined work-related hazardous exposures that women in farmworker families experience, including pesticides [31,42,43,44,45,46,47] and heat [48]. However, few studies have examined the actual organization of work and work safety culture for women in farmworker families. Organization of work includes the processes and organizational practices that influence job design, such as the timing of when work is performed (e.g., shifts and hours worked, seasonality, and flexibility), the physical and psychological demands of work, the decision latitude workers have (e.g., variation in effort and choice in performing work), and style of supervision and support [49,50,51,52]. Work safety culture is the degree to which all members of an organization (management and workers) agree to the value of safety over production [53].

One set of studies has focused on sexual harassment and abuse experienced by women agricultural workers [25,26,27,54,55]. These analyses indicated that sexual harassment is commonly experienced by women working in agriculture. Another set of studies has focused more on the job characteristics of women agricultural workers [43,56,57]. These studies used diverse methods to examine different aspects of work organization and work safety culture. For example, Arcury and colleagues [56] used survey data with 220 Latinx women in farmworker families and found that such organizational dimensions as shift, greater psychological demands, and poorer perceived work safety climate were associated with poorer health indicators. TePoel et al. [57] compared survey results from 31 farmworker couples and found that women had greater work-family conflict and less support at work resulting in moderate stress levels. Curl and colleagues [43] used a mixed-methods approach (survey interviews, focus groups, in-depth interviews) with 70 Latinx women farmworkers and found that major themes included long working hours, concern about pesticide exposure, and limited enforcement of regulations.

Given the lack of information about the organization of work and work safety culture experienced by employed Latinx women in farmworker families, the overall goal of this paper is to document the employment characteristics of Latinx women in farmworker families. Using interview data collected from participants over an 18-month period, this analysis describes (1) job churn among Latinx women in farmworker families; (2) their most recent jobs’ organization of work; (3) the work safety culture experienced by these women in their most recent jobs; and (4) examines differences in job churn, organization of work, and work safety culture between women in farmworker families employed as farmworkers and those with non-farmworker occupations.

## 2. Materials and Methods

The data for this analysis are from the Preventing Agricultural Chemical Exposure 5 (PACE5) study. PACE5 is a long-term (begun in 1996), community-based participatory research program. The main collaborators are Wake Forest School of Medicine (Winston-Salem, NC, USA) and the North Carolina Farmworkers Project (Benson, NC, USA). The overall goal of PACE5 is to delineate the effects of pesticide exposure on child neurocognitive development. The Wake Forest University School of Medicine IRB approved the PACE5 study protocol, and the study received a Certificate of Confidentiality from the National Institutes of Health. Certificates of Confidentiality “protect the privacy of research participants by prohibiting disclosure of identifiable, sensitive research information to anyone not connected to the research except when the participant consents or in a few other specific situations” (https://grants.nih.gov/policy/humansubjects/coc.htm, accessed on 4 April 2022).

### 2.1. Sample

PACE5 recruited a sample of children aged 8 years who had completed the first grade in a US school. This analysis focuses on the PACE5 mothers of those child participants in farmworker families; these are families in which at least one adult member had been employed in agriculture during the three years preceding baseline recruitment in 2017. Inclusion criteria for the families were that they self-identified as Latinx, and had family incomes below 200% of the Federal Poverty Level. Participants were recruited over the period from March 2018 to December 2019 from eastern North Carolina counties with large farmworker populations. Families were excluded from the study if their child had a life-threatening illness, prior history of neurological conditions, physical condition or development disorder that would not allow them to complete or would interfere with the results of neurobehavioral tests or brain imaging (used in the main study). Families were also excluded if a primary language other than Spanish or English was spoken in the home, or if the mother refused to complete the questionnaires.

Based on information provided by the North Carolina Farmworkers Project and our other community partners, bilingual research staff contacted parents and explained the overall study procedures, answered questions, and, if the parent agreed to participate, obtained signed informed consent from the parent and assent from the child. Data for 67 of 76 women in farmworker families were available for this analysis. These 67 women completed a baseline interview and five quarterly follow-up interviews, for six data points. Fifty-nine of the 67 women were employed in the 12 months prior to the completion of their Follow-Up 5 Questionnaire and completed detailed questions about their jobs’ organization of work and work safety culture. Because project staff worked through community partners, the number of potential participants or their parents who refused to participate is not known.

### 2.2. Data Collection

Data are from a baseline questionnaire and up to five follow-up questionnaires (four participants did not complete all five follow-up questionnaires, with three missing one follow-up questionnaire, and one missing two follow-up questionnaires). Follow-up questionnaires were originally scheduled to be conducted on a quarterly basis so that Follow-Up 5 Questionnaires would have been completed June 2019 and March 2020. However, the COVID-19 pandemic disrupted data collection, with no data collection being completed from March through May 2020. Therefore, 13 questionnaires that should have been completed in March 2020 were completed in June through August 2020, with six delayed three or more months, four delayed two months, and three delayed one month. Forty-eight of the Follow-Up 5 Questionnaires were completed before the COVID-19 disruption, and 19 were completed after the COVID-19 disruption.

All questionnaires included items on the women’s employment and occupations, as well as their personal characteristics. The Follow-Up 5 Questionnaire included an extensive list of items on the job characteristics of the women’s most current job, if they had been employed at any time during the 12 months prior to the interview. Questionnaire items were taken from existing questionnaires and scales when possible, particularly from those validated in Spanish. New questionnaire items were developed in English, translated to Spanish, and back translated to English; item wording was adjusted to ensure consistent meaning across languages.

Interviewers were native Spanish speakers; all spoke English, but with varying degrees of proficiency. They completed training before data collection began. The interviewers entered data in real time using Research Electronic Data Capture (REDCap), hosted at Wake Forest School of Medicine through the Clinical and Translational Science Institute [58]. Participants were given a $20 cash incentive for completing the baseline questionnaire, and a $20 cash incentive for completing each quarterly follow-up questionnaire.

### 2.3. Measures

Measures were constructed for participant baseline personal, immigrant and acculturation, family structure, and financial characteristics. Personal characteristics at baseline included age, in the categories 25 to 29 years, 30 to 34 years, 35 to 39 years, and 40 to 45 years. Immigrant and acculturation characteristics included place of birth (Mexico, other Latin American country, US), fluent in English (no, yes), and educational attainment (11 or fewer years, 12 or more years). Family composition and disruption characteristics included marital status (married or living as married, not married), spouse always present in family (no, yes), number of adults in household (1, 2, 3, or more), number of children in household (1 or 2, 3, 4, or more), and number of residential moves in last eight years (0, 1, 2, 3, or more). Financial characteristics included employed outside the home (no, yes), occupation (farmworker, non-farmworker, and not employed outside the home), employed spouse in household (no, yes), food security (high versus other [marginal, low, very low]), financial hardship during the past 8 years (often, rarely, never), and whether they send money to relatives back home (no, yes). Non-farmworker occupations included those in manufacturing, building and grounds cleaning, food preparation, personal care and service, health support, and office and administrative support. Food security was measured with the Spanish-language adaptation [59] of the US Household Food Security Survey Module [60]. Financial hardship in the past eight years was measured with an item from an adverse childhood experiences inventory [61].

Job churn is an indicator of job-to-job movement among workers within a labor market [62]. This movement could include moving from employment outside the home to work inside the home. Job churn measures include the following: (1) number of times employed outside the home at baseline and quarterly follow-up interviews with the values 0 (never employed) to 6 (always employed); (2) number of changes in occupation with the values of 0 to 5, with the understanding that some participants would be given the value of multiple occupations even though they had the same occupation for two different questionnaires, but changed to a different occupation in between these two different questionnaires; (3) number of changes in and out of the work force, with the values of 0 to 4; and (4) number of times employed as a farmworker, with the values 0 (never employed as a farmworker) to 6 (always employed as a farmworker).

Organization of work measures include job structural characteristics, job control, and job content. Job structural characteristics at the Follow-Up 5 Questionnaire included employed in past 12 months (no, yes), employment type (temporary, seasonal, permanent), most recent occupation in the past 12 months (farmworker, non-farmworker), hours worked per week in primary job (fewer than 32, 32–40, more than 40), hours worked per week in all jobs (fewer than 32, 32–40, more than 40), hourly pay in primary job ($7.50 to $8.50, $9.00, $10.00 to $11.00), and whether paid piece-rate (no, yes). Benefits included whether participants receive health insurance, paid vacation, paid sick leave, paid holidays, or a retirement plan. Extra pay includes whether participants received extra pay for working more than 8 h per day, working more than 40 h per week, finishing work early, working on weekends, or working special shifts.

Perceived job control was assessed with three items that asked whether the participants felt that they were able to make decisions about their work schedule, the number of hours worked, or their wages. The three dichotomous items were summed, with higher scores indicating greater perceived job control.

Three aspects of job content were assessed with 11 items from the scale developed by Karasek and colleagues [63]. Items in this scale are scored on a four-point Likert scale with the values “seldom/never” (1) through “almost always” (4). Skill variety is the mean of four items (e.g., “How often does your job require you to learn new things?”, “How often does your job require you to be creative?”) (Cronbach’s *α* = 0.44). Decision latitude is the mean of three items (e.g., “How often are you allowed to make your own decisions about your work?”) (Cronbach’s *α* = 0.80). Psychological demands is the mean of four items (e.g., “How often does your job require you to work very fast?” “How often is your job very hectic?”) (Cronbach’s *α* = 0.75). Each of these measures has been used with immigrant Latinx worker populations [56,64,65].

Work safety culture measures include perceived vulnerability and work safety climate. Perceived vulnerability was assessed with five dichotomous items taken from an instrument developed by Vives and colleagues [66]. These items asked whether during the past 12 months on her main job the participant felt defenseless against unfair job treatment, treated in a discriminatory or unjust manner, afraid to voice a safety concern, afraid of being fired even though she had done nothing wrong, and that she could be easily replaced. The five items were summed and values for the summary score ranged from 0 to 5, with higher scores indicating greater vulnerability. These items have been used in previous research examining the work safety culture of Latinx women [56].

Perceived work safety climate was evaluated with the scale developed by Gillen and colleagues [67]. This scale includes nine four-point Likert items with the values strongly agree (4), agree, disagree, and strongly disagree (1). Items include statements such as “workers’ safety practices are very important to the boss/supervisors,” and “workers receive instructions on safety when hired.” The nine items were summed and values for the summary score ranged from 9 to 36, with higher values indicating better work safety climate (Cronbach’s *α* = 0.86). A tenth item included with the scale (but not in the score) asks participants to rank the degree to which their supervisors care about safety with the values “they do as much as possible to make my job safe,” “they could do more to make my job safe,” and “they are only interested in doing the job fast and cheaply.” This scale has been used in previous research with male and female Latinx workers [68,69,70].

### 2.4. Analysis

For all women in farmworker families, descriptive statistics (counts, percentages, mean, standard deviation [SD], median, interquartile range [IQR], as appropriate) were calculated for baseline participant characteristics of interest, job churn and farmworker employment over time. Within the subset of participants employed in the prior year at the Follow-Up 5 Questionnaire, descriptive statistics were also calculated for farmworker employment status over time, job structural characteristics, job control, job content scales, vulnerability, and perceived work safety climate. Associations between farmworker versus non-farmworker status at the Follow-Up 5 Questionnaire visit and job structure, job content, and work safety climate were tested using Chi-Square, Fisher’s Exact, or Kruskal–Wallis tests as appropriate. *p*-values of less than 0.05 were considered statistically significant.

## 3. Results

### 3.1. Participant Characteristics

The 67 women ranged in age from 25 to 45 years, with most (68.7%) being in their 30s (Table 1). Most were born in Mexico (80.6%) or another Latin American country (14.9%); three (4.5%) were born in the US. Few (11.9%) reported being fluent in English, and most (83.6%) had fewer than 12 years of education. Most (83.6%) were married, with most (78.5%) indicating that their spouse was always present. Most lived in households with two adults (76.1%), with about one-third of these households having one or two children, three children, or four or more children. Fewer than one-third (29.2%) did not experience a residential move, with nine (13.9%) having experienced three or more residential moves.

Most (82.1%) of the women were employed outside the home at the time of their baseline interview. The occupation for the plurality (44.8%) of women was farmworker, with 37.3% having non-farmworker occupations, and 17.9% not being employed outside the home. The spouses of all of those who were married were employed. Almost half (46.3%) had high food security, but 40.3% reported low food security. Over one-third (38.8%) indicated that they often experienced financial hardship, with 20.9% reporting they never had financial hardship. About one-in-five sent money to relatives back home.

The personal, immigration and acculturation, family structure and disruption, and financial characteristics were virtually the same for the total sample of 67 women and the subsample of 59 women employed at the Follow-Up 5 Questionnaire (Supplemental Table S1).

### 3.2. Job Churn

These women reported considerable job churn (Table 2). Three (4.5%) women reported not working at each of the six interviews, and 20 (29.8%) reported working at all six interviews, both indicating no changes in being employed. However, the remaining 44 women report working at the time of two to five of the interviews, indicating movement in and out of employment. Similarly, although ten (14.9%) women never changed their occupation and 12 (17.9%) changed occupation one time, most participants changed occupations two (34.3%) or three (23.9%) times. Six women changed occupations four (6.0%) or five (3.0%) times. About one-third (35.8%) of the women never changed their employment status in or out of the work force (they never worked or always worked), but most women changed their employment status once (23.9%) or twice (29.8%). A few changed in-out of the work force three (9.0%) or four (1.5%) times.

Among the 67 women in farmworker families, 13 (19.4%) were never employed as farmworkers at any of the six interviews, meaning that four in five were employed as farmworkers at some time (Table 3). Two women were employed as farmworkers at all six interviews, with 19.4% employed as farmworkers at five interviews, 11.9% at four interviews, 16.4% at three interviews, 19.4% at two interviews, and 10.5% at one interview. These percentages were comparable among the 59 women employed in the past year at the Follow-Up 5 Questionnaire.

### 3.3. Organization of Work

Fifty-nine of the women had been employed during the 12 months prior to the Follow-Up 5 Questionnaire (Table 4). At the time they were interviewed, 13 of these women (five farmworkers and eight non-farmworkers) were temporarily not employed. Most (78.0%) of the 59 women described their employment as seasonal, with 15.2% describing their employment as temporary, and 6.8% as permanent (Table 4). The most recent occupation for most (62.7%) was farmworker. Most (67.8%) worked 32–40 h per week, with 28.8% working more than 40 h per week in their primary job. When employment in all jobs was considered, only one worked fewer than 32 h per week, and 35.6% worked more than 40 h per week. Most (83%) of the women were paid less than $10 per hour, with $9 per hour being the modal (69.1%) wage rate. Most (62.7%) were paid based on piece-rate.

Irrespective of occupation, the women did not receive any benefits (Supplemental Table S2). One woman reported receiving health insurance, and two reported receiving paid vacation, paid sick leave, paid holidays, and a retirement plan. Similarly, few reported receiving any bonus pay (Supplemental Table S2). Two reported receiving bonus pay for working more than 8 h in a day, four reported receiving bonus pay for working more than 40 h in a week, and two reported receiving bonus pay for working weekends. None reported receiving bonus pay for finishing their work early or for working a special shift.

Few of the women perceived that they had any job control, with only one (1.7%) woman stating that she was able to make any work schedule, hours worked, or wage decisions (Supplemental Table S3). The women felt that their jobs had little skill variety (mean 1.5, SD 0.4; median 1.5, IQR 1.3–1.8), had little decision latitude (mean 1.1, SD 0.3; median 1.0, IQR 1.0–1.0), and were psychologically demanding (mean 2.0, SD 0.6; median 1.8, IQR 1.8–2.3) (Table 5). For example, 62.7% stated that their jobs seldom or never required that they learned new things, while 84.7% noted that their jobs almost always involved a lot of repetitive work. For decision latitude, 91.5% noted that they are seldom or never allowed to make their own decisions at work, with 81.4% indicating that they seldom or never had the freedom to decide how to do their work. Among psychological demands, 70.7% noted that they were almost always required to work very fast, and 89.6% stated that their jobs were almost always very hectic.

### 3.4. Work Safety Culture

Few women perceived that they were vulnerable at work (Supplemental Table S4). No women reported that they felt defenseless against unfair treatment or treated in discriminatory or unjust manner, one (1.7%) woman reported fear to voice safety concerns or fear of being fired through doing nothing wrong, and four (6.8%) stated they were made to feel that they could be easily replaced.

On the other hand, the mean perceived work safety climate was 13.7 (SD 4.5; median 12.0, IQR 12.0–13.0), where the lowest possible score was 9 and the highest possible score was 36 (Table 6). Most of the women strongly disagreed with the individual safety climate statements. For example, 78.0% strongly disagreed with the statement “workers’ safety practices are very important to the boss/supervisors,” and 79.7% strongly disagreed with the statement “workers receive instructions on safety when hired.” For the single item asking how much supervisors seem to care about safety, 76.3% reported that their supervisors value getting the job done quickly and cheaply over getting the job done safely.

### 3.5. Farmworker versus Non-Farmworker

The women employed as farmworkers in their most recent job at the Follow-Up 5 Questionnaire differed significantly from those employed as non-farmworkers in only one personal characteristic—education. None of the farmworkers had completed high school, while nine (40.9%) of those with a non-farmworker occupation had completed high school (*p* < 0.01).

These women also did not differ in the job churn measures for number of times employed outside the home, changes in occupation, or changes in or out of the work force. They did differ significantly in the number of times they were employed as a farmworker (Table 7). Nine (40.9%) of the non-farmworkers had never been employed as a farmworker. Nineteen (51.3%) of the farmworkers had been employed as a farmworker at four or more of the six questionnaire contacts, while just one (4.6%) of the non-farmworkers had been employed as a farmworker at four or more of the contacts. The women differed significantly in one job structural characteristic: more of the farmworkers (27, 73.0%) than non-farmworkers (10, 45.5%) were paid piece-rate.

Those women employed as farmworkers versus non-farmworkers differed in job content and work safety culture measures. The farmworkers had less decision latitude (median 1.0, interquartile range [IQR] 1.0, 1.0) and less psychological demands (median 1.8, IQR 1.5, 2.0), than did those with non-farmworker occupations (median 1.0, IQR 1.0, 1.7; median 2.3, IQR 1.8, 2.5, respectively). The women did not differ significantly for the work safety climate scale. However, many more of the farmworkers (91.9%) than non-farmworkers (50.0%) indicated that their supervisors were interested in doing the job fast and cheaply rather than safely.

## 4. Discussion

The organization of work and work safety culture experienced by employed Latinx women in North Carolina farmworker families can only be described as abysmal. They experience significant job churn, with most moving in and out of employment and changing occupations several times over an 18-month period. Most report that their jobs are seasonal or temporary. Many report working over 40 h per week (remembering that those employed in agriculture are not entitled to an overtime bonus [71]), being paid less than $10.00 per hour, and working piece-rate [72]. Almost none have any paid benefits. Few feel that they have any job control. They report little skill variety or decision latitude, while experiencing high psychological demands. Their jobs are demanding (e.g., fast and repetitive), offer them little leeway in how the work is done, and offer them little opportunity for learning or creativity. They perceive that their workplaces have an extremely poor work safety climate.

Those women who currently are farmworkers versus non-farmworkers differ in most of these organization of work and work safety culture measures. Farmworkers report less decision latitude and psychological demands than non-farmworkers. Farmworkers perceive a lower work safety climate than do non-farmworkers.

A limited sense of vulnerability is the only bright spot among the organization of work characteristics that these women experience. Perhaps the expectation of temporary and seasonal employment, high job churn, and the limited occupational skills that they need to learn for a new job allow them the power to leave difficult employment situations.

These results expand upon current assessments of the work organization and work safety culture experiences of immigrant workers, particularly women immigrant workers. These results also suggest the effects that organization of work and work safety culture may have on the mental health and wellbeing of women immigrant workers.

The seasonal and temporary nature of the jobs held by women in farmworker families is captured by the level of job churn—the number of changes in labor force participation and occupation in which these women were employed over an 18-month period. Although the level of job churn may provide some sense of protection in a stressful workplace, it also makes these women in farmworker families more vulnerable. Women with lower educational attainment, such as the participants in this study, tend to have greater job-to-job and job-to-non-employment turnover (job churn) [73]. This job churn limits any career advancement. It is also one factor that limits their access to paid benefits. As with most US workers in low-paying, seasonal, and temporary jobs, these women in farmworker families receive no benefits, such as health insurance, paid vacation or sick leave, or retirement [56,74].

The job content measures reported by these employed women in farmworker families—skill variety, decision latitude, and psychological demands—are worse than those reported for other Latinx women manual workers in North Carolina, including those employed in manufacturing [75] as well as others employed in agriculture [56]. Similarly, the perceived work safety climate among the women in farmworker families is worse than that reported in other North Carolina research with Latinx workers employed in agriculture [68,76,77], manufacturing [5,78], or construction [12]. It is lower (mean of 13.66 versus 16.77) than that found among another sample of employed Latinx women in farmworker families [56]. Similarly, it is worse than that reported by other research focused on Latinx workers [10,79]. Most of these other studies have focused on adult males. The low sense of vulnerability reported by the women in the current study is similar to that reported in an earlier analysis of employed Latinx women in farmworker families [56].

### 4.1. Implications for Women’s Mental Health and Wellbeing

The organization of work and work safety culture experienced by these employed women in farmworker families has important implications for their mental health and wellbeing. Several analyses have examined mental health among women in farmworker families [37,38,39,40,41,56]. They document that these women experience elevated levels of stress and depressive symptoms. Some of the stress and depressive symptoms result from the immigration and discrimination experiences of these Latinx women who reside in rural areas of a conservative state. These women also have limited access to health services for themselves and for their children [80,81,82,83]. Although Okonji and colleagues [84] conclude that immigrants have lower odds of having depression than do US citizens, this does not appear to be the case for most Latinx women in farmworker families.

One exception to these patterns is an analysis [47] which found that Latinx women in rural farmworker families and in urban non-farmworker families had relatively low levels of depressive symptoms, with the women in urban non-farmworker families having more depressive symptoms than did those in farmworker families. However, the overall percent of Latinx women in this study with elevated depressive symptoms (8.5%) is similar to the 8.1% of US adults whom meet criteria for depression [85].

How the poor organization of work and work safety culture experienced by these specific women in farmworker families affects their mental health and wellbeing is a matter of speculation, given the design of this study. Based on other research with women agricultural workers [25,26,27,54,55], we expect that work organization at their place of employment affects their mental health and wellbeing as a result of sexual and physical harassment and abuse. The high percentage of these women paid piece-rate also suggests consequences for their general health and wellbeing [72].

Results from a few studies document the associations of work organization on the mental health of women in farmworker families. Arcury and colleagues [56] found that the level of their jobs’ psychological demands was directly associated with stress and depressive symptoms, while skill variety and work safety climate were inversely associated with stress. TePoel and colleagues [57] found that women in farmworker families experienced moderate stress levels as a result of less support at work. Similarly, Arcury and colleagues [75] found the Job Content Questionnaire measure of psychological demand was directly associated with stress and depressive symptoms, and skill variety was inversely associated with depressive symptoms; work safety climate was inversely associated with stress. Adding to their employment stresses, these women also largely carry the burden of the domestic labor of childcare, cleaning, and food preparation [28].

### 4.2. Limitations and Strengths

The interpretation of these results should be tempered by understanding the limitations of this research. The sample is small and non-random. The women had to be mothers of an 8-year-old child at baseline, and all were in low-income families (family income below 200% of Federal Poverty-Level). These factors limit generalizability. The women’s 8-year-old children, rather than the women themselves, were the major focus of PACE5, limiting the information collected about the women. The organization of work and work safety culture concepts measured are limited; other concepts may reflect different circumstances. Little information about the women’s health was collected. At the same time, PACE5 is a long-term community-based participatory research project with a history of collaborating with North Carolina Latinx farmworkers and non-farmworker communities, thereby establishing trust in these communities.

## 5. Conclusions

The jobs available to North Carolina Latinx women in farmworker families are limited. These women appear to experience abysmal organization of work and work safety culture in their employment that does not support their health and safety. The issues confronting these women are similar to those confronting minority and immigrant women who are employed in low-wage and low-skill occupations around the globe. Further research and intervention on the organization of work and work safety culture at places that employ immigrant and minority women manual workers, whether in agriculture, manufacturing, or maintenance, are needed. At the same time, sufficient data are available to inform improvements for the occupational health and safety of women working in rural communities.

## Figures and Tables

**Table 1:** Personal, Immigration and Acculturation, Family Structure and Disruption, and Financial Characteristics for Latinx Women in Farmworker Families, North Carolina, 2018–2019 (N = 67).

Characteristics	n (%)
**Personal**	
Age (in years)	
25 to 29	12 (17.9)
30 to 34	27 (40.3)
35 to 39	19 (28.4)
40 to 45	9 (13.4)
**Immigration and Acculturation**	
Place of Birth	
Mexico	54 (80.6)
Other Latin American Country	10 (14.9)
US	3 (4.5)
Fluent in English	8 (11.9)
Educational Attainment	
11 or fewer years	56 (83.6)
12 or more years	11 (16.4)
**Family Composition and Disruption**	
Married or Living as Married	56 (83.6)
Spouse Always Present in Family ^1^	51 (78.5)
Number of Adults in Household	
1	10 (14.9)
2	51 (76.1)
3 or more	6 (9.0)
Number of Children in Household	
1 or 2	22 (32.8)
3	22 (32.8)
4 or more	23 (34.3)
Number of Residential Moves ^1^	
0	19 (29.2)
1	26 (40.0)
2	11 (16.9)
3 or more	9 (13.9)
**Financial**	
Employed Outside the Home	55 (82.1)
Occupation	
Farmworker	30 (44.8)
Non-farmworker	25 (37.3)
Not employed outside the home	12 (17.9)
Employed Spouse in Household (if married) ^2^	55 (100.0)
Food Security	
High	31 (46.3)
Other (Marginal, Low, Very low)	36 (53.7)
Marginal	8 (11.9)
Low	27 (40.3)
Very low	1 (1.5)
Financial Hardship	
Often	26 (38.8)
Rarely	27 (40.3)
Never	14 (20.9)
Send Money to Relatives Back Home ^3^	13 (22.0)

^1^ n = 65. ^2^ n = 55. ^3^ n = 60.

**Table 2:** Job Churn (Employment Changes) from Baseline to Follow-Up 5 Questionnaire, Latinx Women in Farmworker Families, North Carolina (N = 67).

Job Churn	n (%)
Number of Times Employed Outside the Home at Baseline and Quarterly Follow-up Interviews	
0 (never employed)	3 (4.5)
1	0
2	3 (4.5)
3	10 (14.9)
4	12 (17.9
5	19 (28.4)
9 (always employed)	20 (29.8)
Mean (SD ^1^)	4.5 (1.5)
Median (IQR ^2^)	5.0 (4.0–6.0)
Changes in Occupation	
0	10 (14.9)
1	12 (17.9)
2	23 (34.3)
3	16 (23.9)
4	4 (6.0)
5	2 (3.0)
Changes in/Out of Work Force	
0	24 (35.8)
1	16 (23.9)
2	20 (29.8)
3	6 (9.0)
4	1 (1.5)

^1^ Standard deviation. ^2^ Interquartile range.

**Table 3:** Frequency of Employment as a Farmworker for Baseline Sample (N = 67), and Those Employed in the Past Year at Follow-Up 5 Questionnaire (N = 59), Latinx Women in Farmworker Families, North Carolina.

Number of Times Employed as Farmworker	Baseline (N = 67)	Employed in the Past Year at Follow-Up 5 Questionnaire (N = 59)
	n (%)	n (%)
0 (Never employed as farmworker)	13 (19.4)	9 (15.2)
1	7 (10.5)	7 (11.9)
2	13 (19.4)	13 (22.0)
3	11 (16.4)	10 (17.0)
4	8 (11.9)	7 (11.9)
5	13 (19.4)	11 (18.6)
6 (Always employed as farmworker)	2 (3.0)	2 (3.4)

**Table 4:** Job Structural Characteristics of Participants Employed in the Past Year at Follow-Up 5 Questionnaire, Latinx Women in Farmworker Families, North Carolina (N = 59).

Job Structural Characteristics	n (%)
Employment Type	
Temporary	9 (15.2)
Seasonal	46 (78.0)
Permanent	4 (6.8)
Most Recent Occupation	
Farmworker	37 (62.7)
Non-farmworker	22 (37.3)
Hours Worked per Week—Primary Job	
Fewer than 32	2 (3.4)
32–40	40 (67.8)
More than 40	17 (28.8)
Hours Worked per Week—All Jobs	
Fewer than 32	1 (1.7)
32–40	37 (62.7)
More than 40	21 (35.6)
Hourly Pay (Primary Job) ^1^	
$7.50 to $8.50	7 (12.7)
$9.00	38 (69.1))
$10.00 to $11.00	10 (18.2)
Paid Piece-Rate	37 (62.7)

^1^ n = 55.

**Table 5:** Job Content, Latinx Women in Farmworker Families Employed in the Past Year at Follow-Up 5 Questionnaire, North Carolina (N = 59).

Job Content Scales	n (%)
**Skill Variety**	
How often does your job require you to learn new things?	
(Almost) always	0 (0.0)
Often	4 (6.8)
Sometimes	18 (30.5)
Seldom or never	37 (62.7)
How often does your job involve a lot of repetitive work or tasks? *	
(Almost) always	50 (84.7)
Often	3 (5.1)
Sometimes	2 (3.4)
Seldom or never	4 (6.8)
How often does your job require you to be creative?	
(Almost) always	2 (3.4)
Often	1 (1.7)
Sometimes	5 (8.5)
Seldom or never	51 (86.4)
How often does your job allow you to do a variety of different things?	
(Almost) always	1 (1.7)
Often	13 (22.0)
Sometimes	40 (67.8)
Seldom or never	5 (8.5)
Skill Variety Mean (SD ^1^): 1.5 (0.4)	
Skill Variety Median (IQR ^2^): 1.5 (1.3–1.8)	
**Decision Latitude**	
How often are you allowed to make your own decisions about your work?	
(Almost) always	0 (0.0)
Often	1 (1.7)
Sometimes	4 (6.8)
Seldom or never	54 (91.5)
How often do you have the freedom to decide how you do your work?	
(Almost) always	0 (0.0)
Often	0 (0.0)
Sometimes	11 (18.6)
Seldom or never	48 (81.4)
How often do you have a lot of say about what happens on your job?	
(Almost) always	0 (0.0)
Often	0 (0.0)
Sometimes	7 (11.9)
Seldom or never	52 (88.1)
Decision Latitude Mean (SD ^1^): 1.1 (0.3)	
Decision Latitude Median (IQR ^2^): 1.0 (1.0-1.0)	
**Psychological Demands** **^3^**	
How often does your job require you to work very fast? *	
(Almost) always	41 (70.7)
Often	11 (19.0)
Sometimes	5 (8.6)
Seldom or never	1 (1.7)
How often are you asked to do an excessive amount of work? *	
(Almost) always	7 (12.1)
Often	34 (58.6)
Sometimes	11 (19.0)
Seldom or never	6 (10.3)
How often are you given enough time to get your job done?	
(Almost) always	16 (27.6)
Often	29 (50.0)
Sometimes	12 (20.7)
Seldom or never	1 (1.7)
How often is your job very hectic? *	
(Almost) always	52 (89.6)
Often	2 (3.5)
Sometimes	2 (3.5)
Seldom or never	2 (3.5)
Psychological Demands Mean (SD ^1^): 2.0 (0.6)	
Psychological Demands Median (IQR ^2^): 1.8 (1.8, 2.3)	

* Items reverse scored. ^1^ Standard deviation. ^2^ Interquartile range. ^3^ n = 58.

**Table 6:** Perceived Work Safety Climate, Latinx Women in Farmworker Families Employed in the Past Year at Follow-Up 5 Questionnaire, North Carolina (N = 59).

Perceived Work Safety Climate Scale Items	n (%)
Workers’ safety practices are very important to the boss/supervisors.	
Strongly agree	2 (3.4)
Agree	3 (5.1)
Disagree	8 (13.5)
Strongly disagree	46 (78.0)
Workers are regularly made aware of dangerous work practices or conditions.	
Strongly agree	2 (3.4)
Agree	4 (6.8)
Disagree	8 (13.5)
Strongly disagree	45 (76.3)
Workers are regularly praised for safe conduct.	
Strongly agree	3 (5.1)
Agree	2 (3.4)
Disagree	7 (11.8)
Strongly disagree	47 (79.7)
Workers receive instructions on safety when hired.	
Strongly agree	2 (3.4)
Agree	3 (5.1)
Disagree	7 (11.8)
Strongly disagree	47 (79.7)
Workers attend regular safety meetings.	
Strongly agree	2 (3.4)
Agree	1 (1.7)
Disagree	8 (13.5)
Strongly disagree	48 (81.4)
Proper safety equipment is always available.	
Strongly agree	2 (3.4)
Agree	2 (3.4)
Disagree	7 (11.9)
Strongly disagree	48 (81.3)
Workers have almost total control over personal safety. *	
Strongly agree	49 (83.0)
Agree	4 (6.8)
Disagree	1 (1.7)
Strongly disagree	5 (8.5)
Taking risks is not a part of my job.	
Strongly agree	3 (5.1)
Agree	26 (44.1)
Disagree	20 (33.9)
Strongly disagree	10 (16.9)
The possibility of being injured at work in the next 12 months is very likely. *	
Strongly agree	4 (6.8)
Agree	52 (88.1)
Disagree	2 (3.4)
Strongly disagree	1 (1.7)
Work Safety Climate Score Mean (SD ^1^): 13.76 (4.51)	
Work Safety Climate Score Median (IQR ^2^): 12.0 (12.0–13.0)	
How much do supervisors seem to care about your safety?	
They do as much as possible to make my job safe	6 (10.2)
They could do more to make my job safe	8 (13.5)
They are only interested in doing the job fast and cheaply	45 (76.3)

* Items reverse scored. ^1^ Standard deviation. ^2^ Interquartile range.

**Table 7:** Job Content and Work Safety Culture Compared: Latinx Women in Farmworker Families Employed in the Past Year at Follow-Up 5 Questionnaire as Farmworkers versus Non-Farmworkers, North Carolina (N = 59).

Job Structure, Job Content and Work Safety Culture	Most Recent Occupation		
	Farmworker N = 37	Non-Farmworker N = 22	*p*-Value
	n (%)	n (%)	
Job Structure			
Number of Times Employed as Farmworker			<0.01
0	0 (0.0)	9 (40.9)	
1	1 (2.7)	6 (27.3)	
2	9 (24.3)	4 (18.2)	
3	8 (21.6)	2 (9.1)	
4	7 (18.9)	0 (0.0)	
5	10 (27.0)	1 (4.6)	
6	2 (5.4)	0 (0.0)	
Piece-Rate	27 (73.0)	10 (45.5)	0.03
	Median (IQR ^1^)	Median (IQR ^1^)	
Job Content			
Skill variety	1.5 (1.3, 1.5)	1.5 (1.3, 2.0)	0.06
Decision latitude	1.0 (1.0, 1.0)	1.0 (1.0, 1.7)	<0.01
Psychological demands	1.8 (1.5, 2.0) ^2^	2.3 (1.8, 2.5)	<0.01
Work Safety Climate			
Work safety climate (scale)	12.0 (12.0, 12.0)	13.0 (11.0, 18.0)	0.07
	n (%)	n (%)	
How much do supervisors seem to care about your safety?			
They are only interested in doing the job fast and cheaply	34 (91.9)	11 (50.0)	<0.01
They do as much as possible to make my job safe/They could do more to make my job safe	3 (8.1)	11 (50.0)	

^1^ Interquartile range. ^2^ n = 36.

## Data Availability

The data presented in this study are available on request from the corresponding author. The data are not publicly available to protect participant confidentiality.

## Supplementary Materials

The following supporting information can be downloaded at https://www.mdpi.com/article/10.3390/ijerph19084516/s1: Table S1: Baseline and Follow-Up 5 Questionnaire Participant Personal, Immigration and Acculturation, Family Structure and Disruption, and Financial Characteristics for Latinx Women in Farmworker Families, North Carolina; Table S2: Benefits and Extra Pay Received by Latinx Women in Farmworker Families Employed in the Past Year at Follow-Up 5 Questionnaire, North Carolina (n = 59); Table S3: Perceived Job Control, Latinx Women in Farmworker Families Employed in the Past Year at Follow-Up 5 Questionnaire, North Carolina (n = 59); Table S4: Perceived Job Vulnerability, Latinx Women in Farmworker Families Employed in the Past Year at Follow-Up 5 Questionnaire, North Carolina (n = 59).
