# Supplementary material for: The Abysmal Organization of Work and Work Safety Culture Experienced by North Carolina Latinx Women in Farmworker Families
Source: Int J Environ Res Public Health. 2022 Apr 8;19(8):4516. doi: 10.3390/ijerph19084516 (PMC9029169; doi:10.3390/ijerph19084516)
Supplement: Supplementary file 1 [file ijerph-19-04516-s001.zip › ijerph-1608826-supplementary.pdf]

Supplemental Table S1. Baseline and Follow-Up 5 Questionnaire Participant Personal, Immigration and Acculturation, Family Structure and Disruption, and Financial Characteristics for Latinx Women in Farmworker Families, North Carolina.

Characteristics	Baseline N=67	Follow-Up 5 Questionnaire N=59
	n (%)	n (%)
**Personal**		
Age (in years)		
21 to 29	12 (17.9)	11 (18.6)
30 to 34	27 (40.3)	23 (39.0)
35 to 39	19 (28.4)	17 (28.8)
40 to 45	9 (13.4)	8 (13.6)
**Immigration and Acculturation**		
Place of Birth		
Mexico	54 (80.6)	47 (79.7)
Other Latin American Country	10 (14.9)	10 (16.9)
US	3 (4.5)	2 (3.4)
Fluent in English	8 (11.9)	7 (11.9)
Educational Attainment		
11 years or fewer years	56 (83.6)	50 (84.7)
12 or more years	11 (16.4)	9 (15.3)
**Family Structure and Disruption**		
Married or Living as Married	56 (83.6)	48 (81.4)
Spouse Always Present in Family[1]	51 (78.5)	44 (77.2)
Number Of Adults in Household		
1	10 (14.9)	10 (16.9)
2	51 (76.1)	44 (74.6)
3 or more	6 (9.0)	5 (8.5)
Number of Children in Household		
1 or 2	22 (32.8)	18 (30.5)
3	22 (32.8)	19 (32.2)
4 or more	23 (34.3)	22 (37.3)
Number of Residential Moves[1]		
0	19 (29.2)	16 (28.1)
1	26 (40.0)	24 (42.1)
2	11 (16.9)	10 (17.5)
3 or more	9 (13.9)	7 (12.3)
**Financial**		
Employed Outside the Home		
No	12 (17.9)	9 (15.2)
Yes	55 (82.1)	50 (84.8)
Occupation		
Farmworker	30 (44.8)	26 (44.1)
Non-farmworker	25 (37.3)	24 (20.7)
Manufacturing Occupations	11 (16.4)	11 (18.6)
Building and grounds cleaning and maintenance occupations	6 (8.9)	6 (10.2)

Food preparation and serving related occupations	3 (4.5)	3 (5.1)
Personal care and service occupations	3 (4.5)	2 (3.4)
Healthcare support occupations	1 (1.5)	1 (1.7)
Office and administrative support occupations	1 (1.5)	1 (1.7)
Does Not Work Outside the Home	12 (17.9)	9 (15.2)
Employed Spouse (if married)[2]	55 (100.0)	47 (100.0)
Food Security		
High	31 (46.3)	28 (47.5)
Other (Marginal, Low, Very low)	36 (53.7)	31 (52.5)
Marginal	8 (11.9)	8 (13.5)
Low	27 (40.3)	22 (37.3)
Very low	1 (1.5)	1 (1.7)
Adverse Income		
Often	26 (38.8)	23 (39.0)
Rarely	27 (40.3)	24 (40.7)
Never	14 (20.9)	12 (20.3)
Send Money to Relatives Back Home[3]	13 (22.0)	13 (22.0)

[1]n = 65 and n = 57, respectively
[2]n = 55 and n = 47, respectively
[3]n=60

Supplemental Table S2: Benefits and Extra Pay Received by Latinx Women in Farmworker Families Employed in the Past Year at Follow-Up 5 Questionnaire, North Carolina (N-59).

Benefits and Extra Pay	n (%)
Benefits	
Health insurance	1 (1.7)
Paid vacation	2 (3.4)
Paid sick leave	2 (3.4)
Paid holidays	2 (3.4)
Retirement plan	2 (3.4)
Extra Pay	
Work more than 8 hours in a day	2 (3.4)
Work more than 40 hours in a week	4 (6.8)
Finish work early	0
Work on weekends	2 (3.4)
Work special shift	0

Supplemental Table S3: Perceived Job Control, Latinx Women in Farmworker Families Employed in the Past Year at Follow-Up 5 Questionnaire, North Carolina (N-59).

Perceived Job Control	n (%)
Able to Make Any Work Schedule Decisions	1 (1.7)
Able to Make Any Hours Worked Decisions	1 (1.7)
Able to Make Any Wage Decisions	1 (1.7)
Control Score Mean (SD): 0.05 (0.39)	
Control Score Median (IQR): 0.00 (0.00-0.00)	

Supplemental Table S4: Perceived Job Vulnerability, Latinx Women in Farmworker Families Employed in the Past Year at Follow-Up 5 Questionnaire, North Carolina (N-59).

Job Vulnerability	n (%)
Felt Defenseless Against Unfair Job Treatment	0 (0.0)
Treated in Discriminatory or Unjust Manner	0 (0.0)
Afraid to Voice Job Safety Concerns	1 (1.7)
Afraid of Being Fired Though did Nothing Wrong	1 (1.7)
Made to Feel Could be Easily Replaced	4 (6.8)
Vulnerability Score Mean (SD): 0.10 (0.36)	
Vulnerability Score Median (IQR): 0.00 (0.00-0.00)	
